# Neuropsychological Assessment of Difficulties in Reading Spanish: A Cultural-Historical Approach

**DOI:** 10.11621/pir.2021.0404

**Published:** 2021-06-29

**Authors:** Yulia Solovieva, Athanasios Koutsoklenis, Luis Quintanar

**Affiliations:** a Researcher of Tlaxcala Autonomous University, Mexico; b Assistant Professor, Department of Primary Education, School of Education Sciences, Democritus University of Thrace, Greece; c Researcher of Autonomous University of Puebla, Mexico

**Keywords:** Cultural-Historical, neuropsychology, assessment, reading difficulties, Spanish language

## Abstract

**Background:**

This paper argues that Cultural-Historical neuropsychology provides a solid theoretical framework for the assessment of reading difficulties.

**Objective:**

The objective of the paper is to discuss how reading process and reading difficulties are perceived through the prism of Cultural-Historical neuropsychology.

**Design:**

This paper is of a theoretical and methodological nature, directed to practice and research in the field of neuropsychology.

**Results:**

This paper provides an outline of the fundamental concepts and principles of Cultural–Historical theory that are relevant to the assessment of reading difficulties. It provides a blueprint for the assessment of difficulties in reading Spanish via a presentation of data on common mistakes and their relationship to neuropsychological factors.

**Conclusion:**

The crucial role of in-school teaching in facilitating students to avoid developing reading difficulties, or to overcome them, is highlighted.

## Introduction: Reading from the perspective of Cultural-Historical theory

Cultural-Historical theory understands reading as a kind of intellectual activity directed to the cognitive goal of understanding the meaning of text. Reading is never considered in isolation from the process of writing; the two are examined together as they form written speech (Luria, 2002). fie complex understanding of the meaning of text includes transformation and modification of the meaning according to ideas previously assimilated by a subject, or his or her will to be open and receptive to new ideas. Reading and writing cannot be reduced to motor articulation or visual perception processes; instead, they are complex intellectual activities, which include different processes that can be examined through different levels of analysis (Luria, 2002). Complex verbal communication cannot be limited to the understanding of isolated sentences. In Luria’s words, *“the process of understanding a text as a whole is much more complex and has a variable psychological structure”* (Luria, 2002, p. 259). Variability of the psychological structure of reading depends on the reader’s motives, goals, methods, and the level of acquisition of reading and linguistic structures of each language.

On the level of the brain, the process of reading is represented as a complex functional brain system. Such a system may not be reduced to only visual, tactile, or auditory zones, but includes complex and flexible combinations of joint work among different cortical and subcortical levels with a hierarchic organization. Such organization depends on the level of reading mastery and the structural peculiarities of each cultural language. Reading also includes intention, which is expressed in a concrete motive for the whole action of reading as directed to the specific reading objective. Intention is culturally introduced in infancy through meaningful cooperation with adults and directed to understanding written texts (Vygotsky, 1962).

On a psychological level, reading can be understood as a psychological process or activity. For instance, the reading process may be understood as syllabic, oral, semi-oral, or completely visual (Luria, 1976; Elkonin, 1989; Solovieva & Quintanar, 2020). Reading may be understood as the recognition of signs and symbols. Reading may be realized in different languages, and each cultural language has its own complex structure at several levels (phonetic, lexical, grammar, and text production). In reality, reading is all of these complex processes at the same time. Reading is the reconstruction of the oral word according to a written model of these words (Elkonin, 1989). Reading is a transformation of the profound sense of written text according to the reader’s personality, which is different from superficial ordinary word meaning (Luria, 1976). Reading is also a part of the formation of the child’s personality, since the sense of the texts may modify the child’s image of the world (Leontiev, 1983). Therefore, the ultimate conscious goal of the action, from a psychological point of view, might be to read the text and understand its meaning.

The objective of this paper is to discuss how the reading process and reading difficulties are perceived through the prism of Cultural-Historical neuropsychology. This prism includes the concepts of functional system, neuropsychological factor, and neuropsychological syndrome. These concepts allow us to analyze and anticipate specific mistakes related to particular brain mechanisms. The mistakes are also specific for each language, so in this article they are analyzed for the case of Spanish language. Establishing a clear relationship between the understanding of reading as a complex psychological process, its representation on the brain functional level, and analyses of mistakes made during the assessment of reading in Spanish-speaking subjects, is what we have attempted in this article.

## Fundamental concepts of Cultural-Historical neuropsychology applied to the examination of reading

### Functional systems

As a psychological action, reading includes different operations which take part in the content of this action, and normally, as automatized processes, are not reflected on the conscious voluntary level. These operations are:

1. Visual recognition of each letter in written words or integral words (combinations of graphemes).
2. De-codification of the written word into oral or silent words (articulation).
3. De-codification of written sentences into meaningful ideas.
4. Recognition or modification of the lexical meaning of words.
5. Recognition of grammatical structures and combinations.
6. Reflection on and verification of the meaning according to the goal.

All these operations do not develop automatically and do not appear spontaneously at a given chronological age. They are subject to instruction and should be introduced as voluntary and conscientious intellectual actions, first as a joint action between school teachers and children. In line with Galperin (1998), we argue that not only is phonological consciousness necessary for appropriate learning of reading, but also lexical, grammar, and syntactic consciousness (Solovieva & Quintanar, 2019; Solovieva, Rosas, & Quintanar, 2019; Solovieva & Quintanar, 2020).

The consideration of the content of operations which take part in the process of reading allows for the establishment of a relationship with the brain’s action of reading via the application of the concept of a functional system. Luria (1980) argued that each mental function is based on the integrated functioning of different regions of the brain that unite into functional brain systems. Luria (1980) recognized that thelocalization ofhigher mental functions inthehuman cortex isalways dynamic. The concept of dynamic localization originally developed by Luria (1970a, 1980) has been reformulated by the authors into a *systemic*, *dynamic,* and *hierarchical* representation of psychological actions as functional systems (Solovieva et al., 2019). The brain’s representation of the reading action is *systemic* because a series of specific psychophysiological factors take part in the action. It is *dynamic* because it is dependent on different conditions, such as the stage of learning, type of usage, and age. It is also *hierarchic* because different brain cortical and subcortical mechanisms and relations are involved in this process in different ontogenetic periods. These elements, taken together, form the functional system of reading (either silent or oral).

According to Luria’s proposal for the consideration of functional brain organization (Luria, 1973), the reasons for reading difficulties might be related to an insufficient level of functioning of one of three blocks of the brain: the block of general brain activation, the block of processing sensory information of different modalities (visual, auditory, tactile, and spatial), or the block of programming, regulation, and control. Functional difficulty with each functional brain block may be a “neuropsychological” reason for reading difficulties. The positive or negative role of each functional block in the reading process might be established, not according to the answers of the parents and teachers, but according to the results of clinical qualitative neuropsychological assessment (Solovieva & Quintanar, 2016, 2018).

Luria (1973) has stated that no voluntary processes, including movement, can be carried out by only one brain zone or level. Reading processes are a complex intellectual activity, which is also based on voluntary movements. These voluntary movements are explicit during oral reading and are implicit during reading in silence. Luria (1973) wrote:

*… voluntary movement is the basis of the joint work of diverse brain sectors, and if the apparatus of the first block guarantees the necessary muscle tone, without which no kind of coordinated movement would be possible, the apparatus of the second brain block provides the possibility of afferent synthesis, on which systemic basis the movement takes place, so that the third functional brain block ensures that the whole process of the movement and the action according to the corresponding intentions, creates the programs and the motor realization, and guarantees regulation and control of the course of the movements in order to preserve an organized and conscious character”* (p. 123).

A functional system may be stable if the subject accomplishes a high level of learning of the action, or unstable if the subject only happens to start the learning process or encounters any kind of obstacles or difficulties in the process for any reason (Leontiev & Zaporozhets, 2016). A functional system should not be understood as an elementary process which emerges from the brain. A functional system is rather the physiological basis of and for the execution of the actions that the child learns through interactions with others and within a culture in general. It follows that the formation of functional systems and brain factors is not merely biological, but is essentially a cultural and historical process as well (Luria, 1980). According to Luria (1973),

*… each form of conscious activity is always represented as a complex functional system and is fulfilled with the help of joint work of three cerebral blocks, each of which accomplishes its own participation in the realization of the psychological process* (p. 123).

The task of Cultural-Historical neuropsychology is not to provide a diagnostic label (e.g., dyslexia), but to study the origin of the difficulties from the perspective of functional brain organization, and to establish the precise reason for the child’s functional difficulties.

### Neuropsychological factor

A neuropsychological factor *“is a structural-functional unit characterized by a definite principle of psychophysiological activity and functioning (modus operandi)”* (Glozman, 2007, p. 73). The neuropsychological factor is a product of the functional role of a specific brain zone or level, which might be identified in the psychological actions of a subject.Difficulties with reading processes emerge due to the inadequate development or functional state of different types of neuropsychological factors (Torrado, Solovieva, & Quintanar, 2018). No psychological action can be fulfilled by a single neuropsychological factor. At the same time, these neuropsychological factors may contribute to a variety of psychological actions. Each brain factor is related to various brain cortical and subcortical zones, which take part in the realization of the operation (Luria, 1980). The reading process is carried out by the active participation of different neuropsychological factors.

Very frequently, children present the “symptoms” of difficulties for the codification of sounds into letters of the language they study at school. There is no one functional brain reason that explains these difficulties. Plenty of different reasons might underlie this symptom. These various reasons might include difficulties with the phonemic and phonematic analysis of sounds of oral speech, problems with the kinesthetic analysis and synthesis of the motor articulations of oral speech, complications with the visual and spatial analysis of graphic signs, and difficulties with the regulation and control of their own voluntary activity. All these kinds of functional deficits correlate with the child’s struggles in acquiring reading skills. In each case, the child would present specific typical mistakes in the reading process. At the same time, such errors in reading will appear together with similar types of mistakes in the other school activities, such as writing, oral speech, mathematics, drawing, etc. *Table 1* shows the participation of different neuropsychological factors in the process of reading in the Spanish language.

**Table 1:** The neuropsychological factors responsible for reading difficulties and their involvement in reading Spanish

Neuropsychological factors	Manifestation of deficit in reading
Phonematic discrimination	Confusion with sounds according to phonological oppositions; poor lexicon; confusing the meaning of concrete words; a preference for understanding sentences and texts instead of concrete words. A preference for silent reading but only in cases of previous premorbid automatized acquisition of reading. Never observed in school-age Spanish-speaking patients.
Kinesthetic analysis and synthesis	Confusion and substitution of consonant sounds close in mode and posture of motor production/articulation; omission of consonants; omission of consonants in cases of a complex combination of consonants; poor lexicon; misunderstanding of long, new, uncommon words; difficulties understanding sentences and texts; difficulties in all modalities of oral articulation. A preference for short words and sentences. A preference for silent reading and writing but only in cases of previous premorbid automatized acquisition of reading. One of the most common causes of reading difficulties in Spanish-speaking children in primary school. Very poor reading understanding.
Audio-verbal retention	Difficulties understanding long sentences; problems understanding low-frequency words; problems understanding pairs of words similar in oral pronunciation; virtual absence of understanding long texts. Only observed in patients with brain damage, starting from 12 years old.
Spatial simultaneous analysis and synthesis	Substitution and confusion of spatially close letters; difficulties with evoking entire words explicitly; difficulties understanding phrases and sentences with logical (temporal, spatial) prepositions and connectors; difficulties with understanding comparative, genitive, cause–effect with passive mode and subjunctive mode sentences. Severe difficulties understanding sentences with complex grammar structures and texts. Apreference for understanding words and short sentences with a limited number of prepositions. No articulation difficulties. Very poor reading understanding. Very common, starting from 6 or 7 years old due to developmental or acquired functional deficit.
Visual retention	Confusion of similar graphic images such as letters and drawings. Inability to produce any concrete image or sign. Confused understanding and evoking similar words according to a concrete image. Never observed in Spanish-speaking school children with developmental difficulties.
Sequential motor or kinetic motor organization	Omissions, substitutions, confusions of consonants and entire syllables or short words, and perseveration of sounds, syllables, and words. Diffficulties creating and understanding phrases and sentences. A total inability to understand texts or even sentences. Severe difficulties or even lack of ability to acquire reading and writing skills at school. Poor oral speech; reverting to simple words and common phrases. One of the most common reasons for reading difficulties in Spanish-speaking school children.
Programming, regulation, and control	General inability for flexible learning, including reading processes. Anticipation and substitutions of letters, words, phrases, or sentences close in meaning or pronunciation; inability to verify mistakes in reading; lack of interest in reading; absence of critical reflection on messages or instructions. Misunderstanding and inability to understand the meaning of rules and common patterns; lack of guessing or insight during reading. Notable difficulties applying orthography rules. Absence of proper expression and intonation, difficulties following signs of expressions and making pauses during reading aloud. One of the most common reasons for problems in the reading process.
General brain activation, energetic tone of cortical functioning	Difficulties following orthography rules; constant changes in all kinds of mistakes; commonly makes mistakes in the pronunciation of words when reading aloud; lack of interest in reading texts. One of the most common reasons for difficulties in school learning in general and not so evident for the reading process.

According to the conception of a complex functional system, it is possible to identify specific mechanisms or components of reading as psychological processes related to the functioning of certain brain zones. It is important to stress that before identifying the brain mechanisms, it would be necessary to provide a psychological analysis of the content of the reading process. In other words, psychological analysis of the structure and content of the cognitive action might be carried out before the neuropsychological analysis of the functional system and its brain mechanisms. Brain representation of reading actions is dynamic because it’s changeable according to different conditions: stages of learning, type of usage, inner structure (language), age, etc. Brain representation of reading is systemic because a series of specific psychophysiological (cortical and subcortical) mechanisms take part.

The brain might be intact but the existence of the brain without cultural activity would mean the absence of functional systems. A functional system might be understood, not as a feature of the brain itself, but as the basis for executing actions, when the subject (a child) learns within cultural interactions with others, and with the culture in general. According to the belief of Cultural-Historical psychology about the formation of a functional system as active action (Leontiev, 1983; Solovieva & Quintanar, 2020), the absence of cultural interactions leads to a lack of psychological actions (reading) and, at the same time, to a lack of corresponding functional systems.

### Neuropsychological syndrome

Following the principles of Luria (2002), the authors have proposed an understanding of the neuropsychological “syndrome” in child neuropsychology, including different levels of analysis, such as: a) the level of a material (anatomic or neurophysiological) cause of impairment; b) the level of functional brain mechanisms; c) the level of psychological actions, which will suffer as the consequence of the mechanism; and d) the level of speech or linguistic difficulties related to the functional brain mechanism (Solovieva & Quintanar, 2016, 2018). The level of neuropsychological factors may help to establish a relationship between the mentioned levels of analyses, which may be applied to diverse combinations of learning-difficulty symptoms, including reading difficulties. The determination of a specific neuropsychological syndrome may be made based on a qualitative neuropsychological assessment, taking into account specific elements of different functional systems and the psychological age of the child.

A neuropsychological syndrome is not a sum or a combination of different external behavioral symptoms (Solovieva & Quintanar, 2016). Considering that there is the same central factor or brain-function impairment causing such disturbances, the neuropsychological syndrome is always conserving some psychological functions with the disturbances of the other functions. A neuropsychological syndrome would never include only one disturbed function, for example, the reading process. There would always be several functions affected or disturbed by the same neuropsychological factor.

During learning at school, the child only learns psychological processes or acquires intellectual actions (Galperin, 2000; Talizina, 2018). The difficulties appear not only on the level of consolidated processes but in intellectual activities during development or during the teaching–learning processes. These problems might be understood better with the term “obstacles.” The objective of neuropsychology is to understand these obstacles better in order to propose and organize the method for overcoming such obstacles.

The neuropsychological syndrome, in these cases, should be understood as involving specific obstacles for learning some cultural activities (reading), always combined with difficulties for learning other cultural activities (writing, drawing, speech production), based on the same brain-function deficit or the same neuropsychological factor. The cultural actions which do not include this factor, present a positive level of development. The cultural actions which include this factor, present a deficient level of development. The child may or may not be conscious of their own difficulties.

The consequence of such neuropsychological syndromes would always include personal effects in the child’s life, such as poor school learning, negative communication with peers and teachers, absence of broad cognitive interests, and so on. Each concrete case of reading difficulties should be analyzed from different levels or different points of view: 1) the neuropsychological factor, 2) affected and unaffected intellectual actions, and 3) negative consequences in the child’s personality. In the case of reading difficulties, as in any other kind of verbal difficulties, one more level must be included during the analysis of difficulties: the linguistic level. This level comprises specific features or the structural organization of language: for example, phonologic, lexicon, grammar, or syntax at the level of general verbal expression as the sense of communicative expression (Bajtin, 2009).

*Table 2* shows the levels for analysis of neuropsychological syndrome in cases of learning disabilities, according to our proposal. Each concrete case of learning diffficulties should be analyzed according to these levels. The level of neuropsychological factors should unify and explain all the clinical manifestations of the rest of the levels.

**Table 2:** Levels for analysis of the neuropsychological syndrome for cases of learning disabilities

Levels of analysis of syndrome	Content
Neuropsychological	Brain functional mechanisms responsible for difficulties in learning
Psychological (intellectual actions)	School actions which show the proper level of gains and school actions, which suffer according to an established factor
Personality	Effect of learning difficulties on a child’s social relations and interests
Linguistics	Concrete verbal difficulties related to any level of language structure: phonological, lexicon, grammar, syntax, etc.

As shown in *Table 2*, reading difficulties never appear as isolated problems. Brain mechanisms or factors which might be identified as the functional reason for reading difficulties, trigger problems at different levels: psychological, personal, and verbal. The student shows multiple learning difficulties. Even then, only reading difficulties are detected by the teacher. Reading challenges are accompanied by challenges in writing, problem-solving, productive tasks, and so on. The combinations and manifestations of these difficulties differ depending on each neuropsychological factor (*Table 1*). At the same time, the weak functional state of each factor causes typical mistakes by the child in the process of reading, which might be described in detail for each language system.

### Common mistakes in reading Spanish and suggestions for their correction

From a Cultural-Historical neuropsychological point of view, the most crucial aspect of assessing reading difficulties involves the nature and explanation of errors (Ardila, 1992). Finding and identifying reading difficulties is a necessary but first step that simply provides a gross estimation. In the case of functional deficits of neuropsychological brain mechanisms, the effect of weak mechanisms would be necessarily reflected in all intellectual actions and operations of the child. The whole act of reading and understanding of written expression will suffer as a “complex verbal communicative” (Luria, 1998, p. 393). From the perspective of linguistics, the mistakes that appear in the reading process might be related to different levels, such as phonological, morphological, grammar, syntax, and the level of the sense of verbal communication (Bajtin, 2009).

As previously mentioned, the functional system of the reading process includes several functional brain mechanisms. The functional deficit of each brain mechanism provides specific difficulties. Such deficits are related to an unfavorable functional stage of neuropsychological factors, commonly detected as responsible for problems in the reading process in Spanish-speaking school children (Solovieva & Quintanar, 2020). These factors are 1) kinesthetic analysis and synthesis; 2) simultaneous spatial analysis and synthesis; 3) sequential motor organization; 4) programming, regulation, and control; and 5) general brain activation.

It is important to mention that, in the case of reading difficulties in Mexican children (Spanish language), no complications are related to phonematic integration, as was shown in a recent study (Solovieva, Akhutina, Pylaeva, & Quintanar, 2021). Diffficulties pertaining to phonematic integration were reported in studies with reference to adult patients with brain injuries and a diagnosis of sensory aphasia (Pérez, 2000; Solovieva, Chávez, Pérez, & Quintanar, 2001; Benavides, 2015). The authors hypothesize that the phonematic content of Spanish is straightforward. There is no significant quantity of pairs of phonematic oppositions for consonants, as for instance, in Russian and Portuguese (Morais, 2010; Chastinet, et al., 2011, 2012; Morais, et al., 2012). These results contain considerable evidence of the role of the cultural specificity of each language in functional brain participation and in variants of difficulties, which arise during learning reading at school.

1*. Functional deficit in kinesthetic analysis and synthesis* might be observed in constant substitutions and omissions of sound, which are close to the point and mode of articulation production. These reading mistakes are related specifically to the phonological and morphological level of language. The most notable difficulties are related to consonant sounds. These mistakes occur in all kinds of productive oral speech, including repetition of words and sentences or proper individual expressions. For example, children confuse consonant sounds which are similar by mode and place of motor production (articulation), such as “l”, “r”, “ch”, “y”, “d”, “n,” and confuse all kinds of combinations of consonants in words. Similar difficulties appear during reading words, sentences, and texts aloud. Similar omissions also occur in writing by dictating and elaborating independent words, sentences, and texts. These mistakes might not be notable in repetition, especially in the repetition of unique words. Children will also present a very poor lexicon, which might be limited to very few common words, phrases, or sentences. At the same time, children try to articulate entire sentences with all syntactic components: subject, predicate, and direct or indirect object. This kind of difficulty is severe and persistent and reflects constant problems in all oral and written verbal processes. Correction in cases of such challenges is prolonged and requires patience from the therapist, the child, and the parents. Correction should be based on gradual training of precise hand and oral postures with the help of games and actions with objects. Drawings and perceptual table games are useful before the gradual introduction of reading and writing.

2*. Functional deficits in spatial simultaneous analysis and synthesis are* related to understanding and producing long sentences with complex grammar structures, and with severe difficulties with the production and understanding of spatial and temporal prepositions. For example, children do not understand sentences like “The dog runs behind (or in front of) the car.” These reading mistakes are related specifically to grammar and the syntactic level of language. There are no errors in the repetition of words and direct simple sentences or proper short individual expressions, or the inability to produce or understand texts. Technically, reading is accessible but without a proper understanding of written information. Children are able to pronounce long words and sentences. In some cases, confusion between similar letters in a visual image or spatial orientation might be found. Systemic effects of these brain mechanisms seriously affect the learning of mathematics, writing, and sciences, which require orientation on the perceptual level, such as tables, graphics, schemes, maps, and systematic rules. Correction should include external orientation to objects and surroundings, with the help of games and external intellectual actions, and then proceed slowly to the level of orientation in perceptual space.

3*. Functional deficits in the sequential motor organization* will be related to severe difficulties in all reading modalities, and even total inability to read. These reading mistakes are related specifically to the phonological, morphological, and grammar– syntactic levels of language, and a very common inability to produce or understand sentences or phrases, with a clear preference for isolated word articulation and understanding. For example, children produce only isolated short nouns instead of long properly-organized sentences, such as “play” instead of “I like to play every day.” There may be considerable difficulties in understanding and producing phrases or sentences with prepositions and with any kind of complex grammar structures, with frequent omission of complex consonants in words, and a total inability for producing or understanding texts. Technically, reading is not accessible at all. Systemic effects of the functional deficit of sequential motor organization affect all intellectual and practical actions based on organized movements. Correction in such cases is prolonged and difficult, and should include exercises for hand movements in established series, such as playing musical instruments, active games with physical actions, and ongoing drawing activity before passing to the level of written speech.

4*. Functional deficit in regulation and control* may be associated with the superficial articulation of common words and sentences, confusion and anticipation of sounds, words, and even sentences while reading texts. For example, children may read “go” instead of “going” or “play back” instead of “playing basketball.” These reading mistakes are related to morphological and grammar–syntactic levels of language, but primarily to the level of sense reception and transmission of verbal communication as the general meaningful expression, with an absence of the ability to critique their own mistakes. There is a total inability to produce or understand the profound meaning of texts and to replace complex sentences and phrases by direct and simple grammar structures in all tasks for reading and writing, with a poor interest in learning in general. Technically, understanding the reading of texts is not accessible at all. Systemic effects of a functional deficit of regulation and control are reflected in all intellectual tasks, which are new, require new understanding, or follow the goal and proper selection of the solution among two or more options. Correction of such difficulties should include detailed work with an orientation provided by steps, games with explicit rules, and external representation of each rule of strategy for each intellectual action.

5*. A functional deficit in general brain activation* may cause superficial articulation of common words and sentences. There is no specific level of linguistic organization of language observed concerning this factor, and more research should be conducted to determine the relationship between general brain activation and linguistic difficulties. It’s possible to foresee potential problems with the general understanding of written texts due to a lack of stability in cognitive functioning. There are constant fluctuations and an absence of consistency in the execution of reading and all intellectual tasks. Children present an adequate reading of isolated words. There is an absence of consciousness of their own mistakes in situations of frustration or tiredness. Severe difficulties for producing or understanding the profound meaning of long texts might be detected with a much better understanding of short and funny stories. Children may show insufficient interest in learning in general, especially for long monotonous tasks. Technically, the understanding of texts is accessible in a good functional state with interest in the topic. Systemic effects of these difficulties also appear in all intellectual actions, which require a long period of concentration and execution of tasks, and repetition of exact information. Correction of such problems should include work with new and motivating tasks, changes in tasks, and the inclusion of rhythmic and active physical actions and games with a clear explanation of rules. *Table 3* summarizes the relationship between five neuropsychological factors as the reason for difficulties in reading, and the linguistic level of language organization for the Spanish language.

**Table 3:** Neuropsychological factor and linguistic levels

Neuropsychological factors	Linguistic level of language
Kinesthetic analysis and synthesis	Phonological and morphological level.
Simultaneous spatial analysis and synthesis	Grammar and syntax level
Sequential motor or kinetic motor organization	Phonologic, morphologic, grammar, and syntax levels
Programming, regulation, and control	Grammar, syntax, and the level of sensitivity of verbal expression and communication. Mistakes on the phonological and morphological levels may be observed.
General brain activation, energetic tone of cortical functioning	No relation between linguistic levels was determined. Probable difficulties for the formation of sense and intention of verbal expression.

## Discussion

According to the principles presented, neuropsychological analyses might provide much more information and explain neuropsychological mechanisms in reading difficulties. The reading process may be hindered by multiple factors of brain functioning, such as kinesthetic integration, phonological integration, motor organization of sequential, flexible movements, regulation and control, and a general stage of activation. In each case, different kinds of mistakes would be typical in children with difficulties in the reading process. It is imperative to study and compare these typical mistakes in other languages. In Spanish-speaking school children, five functional brain mechanisms were observed as the main areas where difficulties arise during reading acquisition, according to the authors’ clinical practice. These factors are: 1) kinesthetic integration; 2) simultaneous spatial analysis and synthesis; 3) motor organization of sequential, flexible movements; 4) regulation and control; and 5) a general activation stage.

Successful identification of such types of mistakes might help to find the proper ways to develop and correct children’s reading. Modern neuropsychology should not be limited only to labeling students according to diagnostic categories, such as those presented in DSM-V (APA, 2013). Instead, it should be providing practical means for an objective understanding of difficulties in the reading process. Such knowledge may offer proper suggestions for teaching, organization of day-to-day activity, and correction. In this sense, reading-correction programs at school have to be directed not to the training and repetition of exercises for strengthening the reading process, but the gradual strengthening of absent, weak psychological operations with the inclusion of weak neuropsychological factors.

It is also essential to consider that difficulties in the acquisition of reading might develop due to inappropriate teaching methods at school, or the low level of a child’s preparation for school learning. School psychologists, neuropsychologists, and teachers interested in analyzing the process of reading should pay special attention to the development of the symbolic function and general verbal communication at preschool age, the pattern of conformation of voluntary activity, cognitive motivation, and emphatic reflection as essential features of the child’s personality.

Several intervention programs that are rooted in Cultural-Historical neuropsychology have been used to help children overcome their difficulties in reading. Evidence has started accumulating for the effectiveness of such methods in Spanish and Russian (e.g., Akhutina & Pylaeva, 2012; Cadavid-Ruiz, Jiménez, Quijano, & Solovieva, 2019; Glozman, 2011; Torrado, Solovieva, & Quintanar, 2018; Solovieva, Torrado, & Quintanar, 2019). New studies in other languages and cultural situations are needed to elaborate a more precise panorama of problems in reading acquisition at school.

## Conclusions

Luria (2002) has provided a neuropsychological analysis of the process of writing for the Russian language. His methodology was used by the authors of this article as a template to analyze the process of reading in the Spanish language. The present article might serve as an example of assessment and syndromic analysis of developmental and clinical difficulties in Spanish-speaking subjects. Detailed analyses of mistakes related to brain functioning might facilitate a better understanding of the reading process, assessment, correction, and teaching for children of different ages. The limitations of this article are related to the absence of coordinated work between neuropsychologists who are specialists in the assessment and development of reading in different languages. The authors hope that the article might serve as a motive for such kind of novel collaboration.
